# Teens’ Social Media Engagement during the COVID-19 Pandemic: A Time Series Examination of Posting and Emotion on Reddit

**DOI:** 10.3390/ijerph181910079

**Published:** 2021-09-25

**Authors:** Saijun Zhang, Meirong Liu, Yeefay Li, Jae Eun Chung

**Affiliations:** 1 Department of Social Work, University of Mississippi, Oxford, MS 38677, USA; 2 School of Social Work, Howard University, Washington, DC 20059, USA; meirong.liu@howard.edu; 3 Thomas Jefferson High School for Science and Technology, Alexandria, VA 22312, USA; 4 Cathy Hughes School of Communications, Howard University, Washington, DC 20059, USA; jaeeun.chung@howard.edu

**Keywords:** social media, posting behavior, emotion expression, mental health, youth, COVID-19, time series

## Abstract

Research has rarely examined how the COVID-19 pandemic may affect teens’ social media engagement and psychological wellbeing, and even less research has compared the difference between teens with and without mental health concerns. We collected and analyzed weekly data from January to December 2020 from teens in four Reddit communities (subreddits), including teens in r/Teenagers and teens who participated in three mental health subreddits (r/Depression, r/Anxiety, and r/SuicideWatch). The results showed that teens’ weekly subreddit participation, posting/commenting frequency, and emotion expression were related to significant pandemic events. Teen Redditors on r/Teenagers had a higher posting/commenting frequency but lower negative emotion than teen Redditors on the three mental health subreddits. When comparing posts/comments on r/Teenagers, teens who ever visited one of the three mental health subreddits posted/commented twice as frequently as teens who did not, but their emotion expression was similar. The results from the Interrupted Time Series Analysis (ITSA) indicated that both teens with and without mental health concerns reversed the trend in posting frequency and negative emotion from declining to increasing right after the pandemic outbreak, and teens with mental health concerns had a more rapidly increasing trend in posting/commenting. The findings suggest that teens’ social media engagement and emotion expression reflect the pandemic evolution. Teens with mental health concerns are more likely to reveal their emotions on specialized mental health subreddits rather than on the general r/Teenagers subreddit. In addition, the findings indicated that teens with mental health concerns had a strong social interaction desire that various barriers in the real world may inhibit. The findings call for more attention to understand the pandemic’s influence on teens by monitoring and analyzing social media data and offering adequate support to teens regarding their mental health wellbeing.

## 1. Introduction

The outbreak of the COVID-19 pandemic has resulted in about 228 million infected cases and 4.7 million deaths [1]. The pandemic has not only compromised the economy and altered people’s lifestyles due to lockdowns and various safety measures but also impaired people’s psychological wellbeing [2]. Teens may be especially vulnerable to the impact because they are in a developmental stage that is particularly sensitive to environmental stress [3]. Teens are characterized by prevalent depression and anxiety and are at a high risk of suicidality, and the pandemic has elevated these risks [4,5].

Social media has become an integral part of teens’ daily life [6]. Social media use has become even more pronounced during the pandemic because of heightened concerns and social distancing [7,8]. Researchers have used data from social media, including Twitter or Reddit, to investigate communications in response to the pandemic [9,10,11,12]. However, few of these studies have particularly focused on teens, and less have compared the responses between teens with and without mental health concerns. In addition, given the evolving nature of the pandemic, it is also important to observe and understand its influence over an extended period.

This study used data from a popular social media platform Reddit [13] to examine teens’ posting behavior and emotion expression concerning significant pandemic events from January to December in 2020 while comparing the difference between teens with and without mental health concerns.

### 1.1. Pandemic and Teens’ Psychological Wellbeing

The COVID-19 pandemic has resulted in prevalent fear, stigma, frustration, stress, and other psychological adversaries worldwide [14,15]. A survey by the US Census Bureau showed that the prevalence of anxiety or depression symptoms in December 2020 was nearly four times as high as that in the previous year. A national study in the UK showed that the prevalence of depression symptoms in June 2020 was twice as high as that before March 2020 when the World Health Organization (WHO) declared COVID-19 a pandemic [2].

The pandemic can be especially devastating to teens because they are susceptible to environmental stress [3]. School closures and virtual learning in the early stage of the pandemic had distanced youth from peers, teachers, and other close people and had stripped their opportunities of extracurricular activities. Recent studies showed that more than one-third of adolescents reported a high level of loneliness during the early stage of the pandemic, a significant risk factor for depression and anxiety [4,5]. The extended homestay may also increase teens’ risks of experiencing familial conflicts and missing needed mental health services [16,17]. As a result, teens during the pandemic are experiencing poorer mental health [17], including anxiety [18,19,20], depression [18,19], and other psychological distress [21]. Nearchou et al.’s study (2020) showed that 22.6% to 43.7% of youth reported depression symptoms during the pandemic, 18.9% to 37.4% had anxiety symptoms, and over half (62.2%) expressed concerns about infection and the health of family members and friends.

Although many studies have examined the pandemic’s influence on teens’ psychological wellbeing, they have primarily relied on survey or interview data. Limited studies have specifically examined teens’ psychological wellbeing that may be reflected through their social media engagement, such as their posting behavior and emotion expression on social media.

### 1.2. Social Media Engagement during the Pandemic

Social media broadly refer to social network and micro-blogging sites represented by Facebook and Twitter, peer production communities represented by Wikipedia, content sharing and discussion forums represented by Reddit, and online dating sites, although these platforms have been converging across these various activities [22]. Social media have become a ubiquitous presence in teens daily life. A recent national survey in the US showed that almost half of the teens from 13 to 17 years old were online “almost constantly”, and nearly all (97%) of them used at least one type of social media [6].

Various theories have been used to understand individuals’ social media engagement. Social capital theories suggest that social media provide important platforms for information exchange and social interactions with minimal geographical and temporal boundaries, which contributes to social capital development and individual wellbeing [22]. Social exchange and social cognitive behavior theories are often employed to explain underlying motivations for social media engagement [23,24,25]. Oh and Syn (2015) [25] tested a framework of motivation and identified learning and social engagement as the most prominent causes for social media interactions. These factors are also correlated with altruism and reciprocity, enjoyment and self-efficacy, community interest, and personal gain. Heaney and Israel’s (2008) [26] social networks–health theoretical framework suggests that social networks can affect individual mental health via influencing stress coping schemes and other mechanisms. These theories jointly indicate that social media are critical venues for health information communication and mutual support and that social media engagement may reflect and influence users’ psychological wellbeing. This may be particularly prominent in the era of the COVID-19 pandemic, given its prolonged social distancing and other safety measures [7].

Empirical research has shown that social media are critical for seeking and sharing health information, obtaining support, and making health decisions [27]. For example, Ngien and Jiang (2021) [28] recently surveyed a sample of young adults during the pandemic and found that social media use can alleviate individual stress by reducing the sense of uncertainty and fatalism through information sharing and mutual support. Schønning et al.’s (2020) [29] systematic review study suggests that social media use, particularly the frequency and duration, is related to various aspects of psychological wellbeing. Researchers have also examined how social media users may respond to health-related information and contexts. For example, Silberman and Record (2021) [30] conducted an experimental intervention study to show that colleague Redditors may respond to a posted message concerning smoke-free policy compliance actively and diversely.

Some studies have used social media data to explore how social media engagement may be related to users’ psychological wellbeing in the pandemic environment. For example, using Reddit data from January to April in 2020, Low et al. (2020) [10] found that a posting spike on a mental-health-related subreddit (i.e., r/HealthAnxiety) in January was about two months earlier than those on other subreddits. This suggests that people with anxiety may be more sensitive to the pandemic. The study also showed that the number of COVID-19-related posts fluctuated along with major COVID-19 events and that posts on mental-health-related subreddits exhibited more negative sentiment than those on other subreddits. Comparing Twitter posts in the US from March to May in 2020 with those from the same period in 2019, Saha et al. (2020) [11] found the expression of mental health symptoms first increased after the pandemic outbreak, then declined, and finally plateaued. Using Twitter data from January to April in 2020, Valdez et al. (2020) [12] identified a significant increase in Twitter volume and elevated sentimentality right after the pandemic outbreak, but both declined gradually later. Recently, MacKay et al. (2021) [31] conducted analyses on the quality and content of Canadian public health and news media’s crisis communication on Facebook. The findings revealed key guiding principles such as a call for action and a conversational tone for effective COVID-19-related crisis communication. In addition, they suggested the importance of building trust to facilitate communication and promote a positive sentiment in the communication. The above-mentioned studies have provided a unique perspective to understand the pandemic’s influence on individual wellbeing, often indicated by users’ posting activities and emotion expression. However, few of these studies have focused explicitly on teens.

### 1.3. Current Study

Despite many studies examining the pandemic’s influence on social media engagement, few have focused explicitly on teens, who may be especially susceptible to the influence [3]. Many of these studies were conducted soon after the pandemic outbreak. It is crucial to extend the observation period further to understand the pandemic’s influence along with its evolution. In addition, although studies have examined the difference between mental health communities and non-mental health communities [32,33], few have examined whether teens with and without mental health concerns differ when they are in the same online community. This knowledge is important for mental health screening and intervention consideration.

Building upon extant research, the current study used data from four Reddit communities (subreddits) to explore teens’ posting behavior and emotion expression in response to COVID-19 over the period in 2020. Specifically, the exploratory study aimed to answer the following research questions (RQs):

**RQ1.**  *How is a teen’s participation in r/Teenagers and the three mental health subreddits related to significant pandemic events over time, and what is the difference between teens in r/Teenagers and teens in specialized mental health subreddits?*

**RQ2.**  *How are teens’ posting/commenting frequencies related to significant pandemic events over time, and what is the difference between teens in r/Teenagers and teens in specialized mental health subreddits?*

**RQ3.**  *How is teens’ emotion expression related to significant pandemic events over time, and what is the difference between teens in r/Teenagers and teens in specialized mental health subreddits?*

**RQ4.**  *Are there differences in posting frequency and emotion expression on r/Teenagers between teens who participated and did not participate in mental health subreddits?*

## 2. Materials and Methods

### 2.1. Data

The study used data from Reddit’s four subreddits, including r/Teenagers, r/Depression, r/Anxiety, and r/SuicideWatch, from 1 January to 31 December 2020. Reddit is one of the most popular social networks worldwide, with more than 430 million monthly active users and over 1000,000 active communities [13]. Reddit users (Redditors) were predominantly in the US (49%) and other western countries (23% of the users were in the UK, Canada, Australia, and Germany). In addition, more than one-fifth of Redditors are youth younger than 19 years old [34].

Reddit allows Redditors to share information and invite comments anonymously and is often used as a social feed of information broadcasted from people’s contacts and audiences [35]. Reddit also allows Redditors with shared interests to form subreddits to focus on specialized content, such as depression, anxiety, and other psychological problems. This helps identify the target population. Unlike survey data or other research data, social media data such as Reddit data usually do not contain participants’ demographic information or other characteristics information for sample identification. By using subreddits’ focuses, researchers can identify a target population such as teenagers and individuals with specific mental health concerns, although this method may somewhat compromise the precision [32,33,36,37]. For example, Park and Conway (2018) compared the posts/comments of r/Depression and other mental health subreddits with those of non-mental health subreddits. The findings revealed that Redditors of the mental health subreddits had more written-communication challenges than other Redditors. In addition, Reddit allows up to 40,000 characters for a post/comment compared with 140 characters on Twitter. This feature, along with its anonymity, makes Reddit an especially suitable platform for discussing mental health topics since such topics often involve extensive information exchange, and stigma can be a concern in the discussion when anonymity is not available.

Following a previous research strategy to identify the target research population on Reddit [32,33], we used r/Teenagers to identify teens as the target research population because it claims itself as “the biggest community forum run by teenagers for teenagers” (reddit.com). A participant who ever posted/commented on r/Teenagers in 2020 were included in the study. Furthermore, we identified participants on the r/Teenagers subreddit who had ever posted/commented on r/Depression, r/Anxiety, and r/SuicideWatch in 2020 as teens who had mental health concerns. The three mental health subreddits were selected because they were the most popular mental health subreddits, and these concerning symptoms were closely related to the pandemic’s influence [4,5,19].

We used Pushshift [38] and PRAW (Python Reddit API Wrapper: https://github.com/praw-dev/praw (accessed on 10 January 2021)) application programming interfaces to download all posts and comments on four subreddits (r/Teenagers, r/Depression, r/Anxiety, and r/SuicideWatch) in 2020. Two separate Reddit accounts were used simultaneously to double the download speed, and the download was completed in a course over 15 days.

The r/Teenagers subreddit has about 2.4 million members for comprehensive topics. The downloaded data included 1,933,065 posts and 17,022,011 comments from the r/Teenagers subreddit; 239,407 posts and 623,233 comments from the r/Depression subreddit; 95,427 posts and 366,769 comments from the r/Anxiety subreddit; and 159,933 posts and 538,986 comments from the r/SuicideWatch subreddit. Each post/comment’s id, timestamp, username, text body, and title were extracted for the analysis. Posts and comments by Redditors who had since deleted their accounts were removed, as they did not contain any content. All posts/comments from r/Teenager in 2020 were included in the analyses. For posts/comments in the r/Depression, r/Anxiety, and r/SuicideWatch subreddits, only those with a username matched to that in r/Teenagers were included in the analysis. A total of 3.5% of teens on r/Teenagers ever participated in one of the three mental health subreddits in 2020.

The study used publicly available Reddit data that did not contain any identifiable information and was exempt from IRB oversight.

### 2.2. Measurement

**Figure 1:**
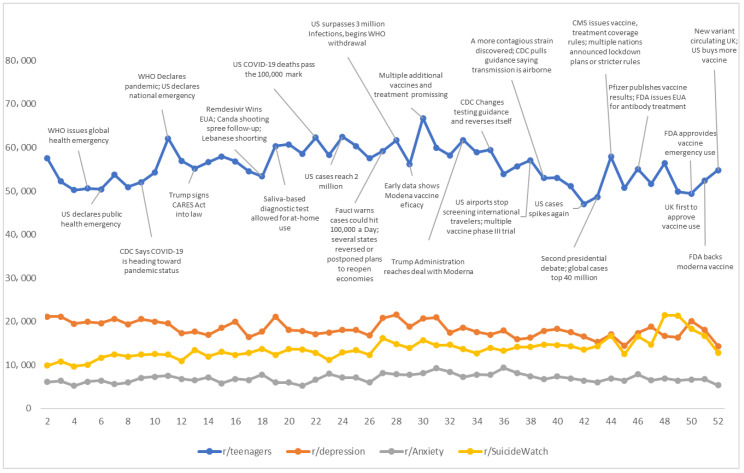
Weekly total number of active participants by subreddits. Note. The first and last weeks were excluded because of incomplete weekdays. The numbers of participants for the three mental health subreddits (r/Depression, r/Anxiety, and r/SuiscideWatch) were multiplied 30 times.

Weekly total number of subreddit participants. Each Redditor’s weekly participation, as reflected by their posts and comments, in each of the four subreddits was indexed and counted. If a Redditor had more than one post or comment in a week, the Redditor would only be counted as 1 participant to calculate the weekly total number of participants. The weekly number was calculated throughout 2020 for r/Teenagers and the three mental health subreddits. Only participants with a username matched to that on r/Teenagers would be counted for the three mental health subreddits. Because the first and last weeks in 2020 were not complete, they were excluded and resulted in a total of 52 weeks for the analysis. Because the numbers of weekly teen participants on the three mental health subreddits were much smaller than those on r/Teenagers, we multiplied the former by 30 times for easy figure display (see Figure 1).

Weekly mean posts/comments per participant. This measure was constructed by dividing the total number of posts and comments by the number of unique active participants in each week.

Negative emotion. We used another python package Text2Emotion (Detecting emotions behind the text: https://shivamsharma26.github.io/text2emotion/ (accessed on 1 February 2021)) to annotate emotions for each post and comment. At first, the python package Text2Emotion was applied to remove any unwanted text, such as stop words, replace shortcuts and contractions with the whole words, and replace emojis with the corresponding words. Next, the package was applied to detect and annotate five emotions (i.e., anger, fear, happiness, sadness, and surprise) for each word in a post or comment by looking it up in a dictionary that mapped words to emotions. If the word was not present in the dictionary, the program skipped to the next word; if it was present, the program found the emotion corresponding to the word and added 1 to the number of words corresponding to a specific emotion. Text2Emotion then calculated the total number of words with a corresponding emotion in a post or comment. The number was then divided by the total number of words in the post or comment and returned five values (range = 0 to 1) corresponding to the five types of emotions, with higher values indicating stronger emotions (see Table 1 for illustration).

To construct one composite score for emotion, we dropped the “surprise” emotion because it may indicate both positive and negative emotions. We then reverse coded the happiness emotion and added its score to the sum of the other three scores (angry, sad, and fear). The compound scale indicated the intensity of negative emotion (see Table 1).

### 2.3. Data Analysis

After the Reddit data were downloaded, statistical software package SAS [39] was used for data treatment and analyses, including matching usernames between r/Teenagers and the three mental health subreddits, identifying and counting active and unique participants on a weekly basis, calculating the mean weekly number of posts/comments per participant, calculating the mean level of emotion, and relevant group analyses. We also visualized the time series data outcomes.

As noted above, we used the python package Text2Emotion to annotate emotions for each post and comment. We then used SAS to summarize mean weekly emotion levels across subreddits.

To observe the relationship between the pandemic evolution and post behavior or emotion, we incorporated the pandemic timeline information [40] when evaluating the posting behavior or emotion. Two authors independently reviewed the pandemic timeline to determine significant pandemic relevant events. The pandemic timeline is presented in Figure 1.

Finally, we conducted the Interrupted Time Series Analysis (ITSA) to examine how posting and emotion before and after the pandemic outbreak on the r/Teenagers subreddit differed between teens with and without mental health concerns. ITSA is a powerful analysis strategy that deals with time series data while handling trend shifting due to an interruption or intervention. In an ITSA study design, a segmented regression approach was used to analyze time series data during the pre- and post-interruption phases [41]. We used the SAS ITSA Macro developed [41] for the analysis, which allows multi-group time series analyses; it also contains diagnostic tools to assess autocorrelation (the correlation of repeated measures over time) in time series data for model selection. The interrupted time series analysis in the ITSA macro is accomplished using Ordinary Least Squares regression with Newey–West autocorrelation adjusted standard errors [41].

## 3. Results

### 3.1. How Is a Teen’s Participation in r/Teenagers Related to Significant Pandemic Events over Time, and What Is the Difference between Teens in r/Teenagers and Teens in Specialized Mental Health Subreddits?

Figure 1 shows the weekly total number of active participants who had at least one post/comment in a specific week across subreddits. The mean number of weekly participants for the r/Teenagers subreddit was 55,794 (range = 47,014–66,744) throughout the year. When observing the pandemic timeline and other events, we found that the weekly total number of participants fluctuated along with the significant pandemic events in 2020. For example, the weekly participants on the r/Teenagers subreddit had increased since week seven and spiked in week 11. These changes corresponded to the US declaration of COVID-19 as a public health emergency (week 7), many passengers on a California cruise ship being tested positive for the COVID-19 virus (week 10), the WHO declaration of COVID-19 as a pandemic (week 11), and the US declaration of it as a national emergency (week 11). Other significant COVID-19-related events were also related to a sudden increase in active participants in the r/Teenagers subreddit. Still, the change magnitude was milder than that in week 11 when the pandemic outbreak was declared. For example, Figure 1 shows an increase in participants in week 24 when the US reached a milestone of 2 million cases and then in week 27 when it was estimated that the US could hit 100,000 cases a day. Positive news also brought an increase in active participants in r/Teenagers, such as weeks 18 and 19 (news of emergency use authorization for Remdesivir and saliva-based diagnostic test for at-home use) and weeks 29 and 30 (news of promising vaccines and treatments). Similarly, the spike in week 44 corresponded to multiple COVID-19-related events, such as the US Center for Medicare & Medicaid Services (CMS) issuing vaccine and treatment coverage rules and numerous nations (including Italy, France, Germany, and the UK) announcing lockdown plans or strict rules for controlling the pandemic.

The participant fluctuation in r/Teenagers after week 45 was also accompanied by significant pandemic-related events, such as the reports of the Pfizer vaccine’s high protection efficacy and the spread of a dangerous COVID-19 variant in the UK. However, social media engagement in this period may be confounded by news reports concerning political struggles following the US election day on November 3 (week 45). Weekly participants in r/Teenagers largely declined from weeks 30 to 42 (late July to mid-October). The period may primarily correspond to the summer and its extension because many schools delayed the fall semester opening in 2020.

The weekly active participants were 609 (range = 478–719) for r/Depression, 230 (range = 173–311) for r/Anxiety, and 456 (range = 322–714) for r/SuicideWatch. The weekly participants for the three mental-health-related subreddits were generally stable throughout the year, except weekly active participants for r/SuicideWatch and r/Depression had a noticeable decline in December, which was the opposite of r/Teenagers.

### 3.2. How Are Teens’ Posting/Commenting Frequencies Related to Significant Pandemic Events over Time, and What Is the Difference between Teens in r/Teenagers and Teens in Specialized Mental Health Subreddits?

**Figure 2:**
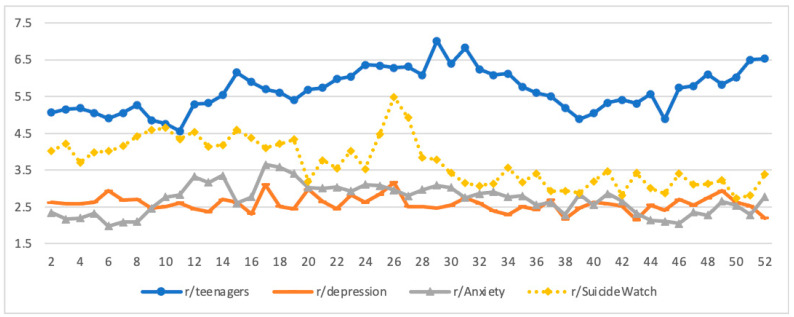
Weekly mean number of posts/comments per participant by subreddits.

Figure 2 shows the weekly numbers of posts/comments per participant, namely weekly posting/commenting frequency, across the subreddits. Overall, teens in r/Teenagers had a higher posting/commenting frequency (*M* = 5.67; range = 4.56–7.01), followed by teens in r/SuicideWatch (*M* = 3.71; range = 2.74–5.49), r/Anxiety (*M* = 2.71; range = 1.98–3.65), and r/Depression (*M* = 2.58; range = 2.15–3.16). The weekly posting/commenting frequency fluctuated substantially for those on the r/Teenagers and r/SuicideWatch subreddits but only moderately for those on the r/Anxiety and r/Depression subreddits. For participants on r/Teenagers, the weekly posting/commenting frequency slightly declined from week 9 to week 11 (see Figure 2). In contrast, the number of weekly active participants spiked during the same period (see Figure 1). From week 11 to 15, the weekly posting/commenting frequency increased from 4.56 to 6.15 posts/comments (35% increase) per participant and remained at around the same level for many following weeks (see Figure 2). However, the weekly active participants during this period declined substantially (see Figure 1). Therefore, it appeared that the increase in weekly participants in r/Teenagers might be negatively associated with a decline in the weekly posting/commenting frequency.

From week 11 (late February) to week 31 (late–July), the weekly posting/commenting frequency in r/Teenagers gradually increased from 4.56 to 6.82 (50% increase) posts/comments per participant, then gradually declined to 4.55 (50% decline) by week 39 (late-September), and then turned around and increased to 6.52 (50% increase) by the end of the year. Week 11 was when COVID-19 was declared a pandemic by the WHO and a national emergency in the US (see Figure 1). The decline from the peak around week 31 corresponded to reports of multiple promising vaccines and treatments. The weekly posting/commenting frequency decrease was primarily accompanied by a reduction in the weekly number of active participants during this period (see Figure 1 and Figure 2). The next increasing trend starting in week 39 also corresponded to pandemic-related events such as CMS rule announcement, multiple nations’ lockdowns and safety measures, and new COVID-19 variants. However, Reddit engagement in this period may be confounded by a series of significant presidential election-related events (e.g., the first and second election debate, Election Day, and political disputes about the election afterward) in the US.

The weekly posting/commenting frequency in r/SuicideWatch also fluctuated substantially, but the pattern differed from that of r/Teenagers. For example, from week 4 to week 10, the weekly posting/commenting frequency for those in r/SuicideWatch increased from 3.71 to 4.65 (25% increase) posts/comments per participant. It then declined to about four posts/comments per participant for several weeks (Figure 2). In contrast, the weekly posting/commenting frequency in r/Teenagers either had few changes or changed in the opposite direction. The weekly posting/commenting frequency in r/SuicideWatch also fluctuated more substantially than that in r/Teenagers. For example, the number in r/SuicideWatch increased from about 3.53 to 5.49 (56% increase) posts/comments per participant from week 24 to 26 and then declined to 3.14 (43% decline) posts/comments by weekly 31, but the changes in r/Teenagers were much milder during the same period.

For participants in r/Anxiety, during the period from weeks 8 to 17, the weekly posting/commenting frequency increased from about 2.1 to 3.65 (74% increase) posts/comments per participant, then gradually declined to 2.28 (38% decline) posts/comments by week 38, and then remained in the range of 2 to 3 posts/comments for the rest of the year. For participants in r/Depression, the weekly posting/commenting frequency was generally stable and in the range of 2 to 3 posts/comments per participant throughout the year, although there were some occasional spikes.

### 3.3. How Is Teens’ Emotion Expression Related to Significant Pandemic Events over Time, and What Is the Difference between Teens in r/Teenagers and Teens in Specialized Mental Health Subreddits?

**Figure 3:**
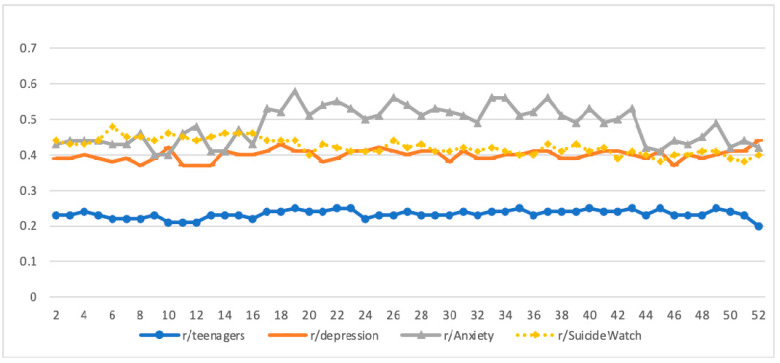
Weekly negative emotion levels in posts/comments by subreddit.

Figure 3 displays the weekly intensity of negative emotion reflected in the posts/comments across the subreddits. Overall, participants’ weekly negative emotion on the three mental health subreddits *(M* = 0.40 to 0.48; range = 0.37–0.58) was substantially higher than that in r/Teenagers (*M* = 0.23; range = 0.20–0.25). The emotion scores for teens in r/Teenagers were generally stable throughout the year. The emotion scores for teens on the three mental health subreddits fluctuated more substantially than those on the r/Teenagers, especially for teens on the r/Anxiety subreddit. For teens on the r/Anxiety subreddit, the emotion scores ranged from 0.4 to 0.47 before week 17, then jumped to 0.53 in week 17 and remained between 0.5 and 0.57 until week 43. The emotion score on the r/Anxiety subreddit had a sharp drop in week 44, and then the scores remained largely under 0.5 throughout the rest of the year. The spike of the emotion score for the r/Anxiety subreddit in week 17 corresponded to the time of the shooting sprees in Canada and Lebanon and subsequent news reports. The substantial decrease in the emotion score in week 44 for r/Anxiety may be related to positive news regarding the pandemic, such as CMS’s vaccine and treatment coverage rules and lockdown and safety measures in multiple nations. The gradual increase in the emotion scores for r/Anxiety from week 45 to 49 corresponded to the US presidential election on November 3 (week 45) and subsequent political disputes during the period. The emotion scores declined after week 49, which corresponded to the settlement of multiple election disputes in the court, the confirmation of the US presidential election results in week 51, and the Thanksgiving break in week 52.

### 3.4. Are There Differences in Posting Frequency and Emotion Expression on r/Teenagers between Teens Who Participated and Did Not Participate in Mental Health Subreddits?

**Figure 4:**
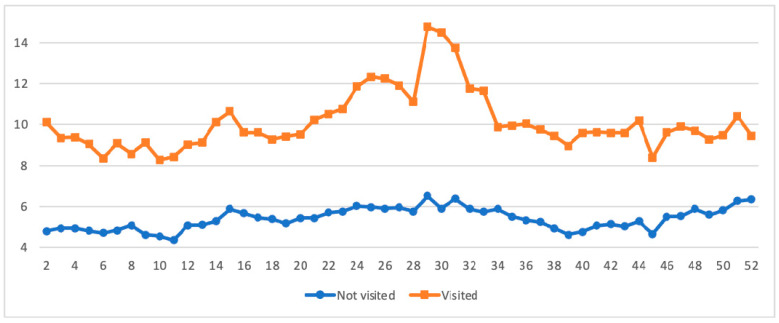
Weekly mean number of posts/comments per participant made in the r/Teenagers subreddit by youth who visited and never visited the mental health subreddits.

Figure 4 displays the weekly posting/commenting frequency in the r/Teenagers subreddit between teens who visited and never visited any of the three mental health subreddits. Teens who ever visited one of the three mental health subreddits in 2020 *(M* = 10.13; range = 8.27–14.78) posted/commented twice as many times compared to those who never visited the mental health forums (*M* = 5.40; range = 4.35–6.52), and the former was also much more fluctuating over time.

**Figure 5:**
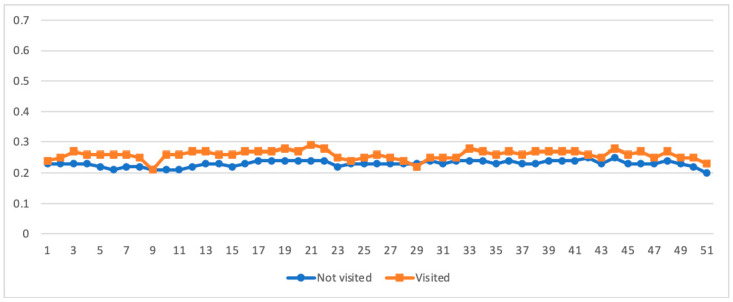
Weekly negative emotion levels on the r/Teenagers subreddit by youth who visited and never visited the mental health subreddits.

Figure 5 shows and compares weekly emotion scores based on posts and comments from the r/Teenagers subreddit between teenagers who visited and never visited any of the three mental health subreddits. Although the difference between teens who visited *(M* = 0.41; range = 0.35–0.45) and never visited (*M* = 0.39; range = 0.35–0.42) the mental health subreddits was minor, the distinction between them was generally clear, and the fluctuation of the former was more considerable.

Table 2 left panel shows results based on the ITSA model that predicated weekly mean numbers of posts/comments while comparing teens with and without mental health concerns. Week 11 was treated as the interruption point when the WHO declared COVID-19 as a pandemic, and a 12-week lag was used in the model to account for the autocorrelation based on the diagnosis results (not shown). The model has a good fit and explained 86% of the variance in the outcome variable (R-square = 0.855). Before the pandemic, there was a declining trend in the mean weekly posting number (b1 = −0.028, *p* = 0.002). However, for teens without mental health concerns, the level (interception) of posting during the post-outbreak phase became higher than the pre-outbreak phase (b2 = 0.741, *p* = 0.002), and the trend reversed from declining to increasing (b3 = 0.032, *p* = 0.03). During the pre-pandemic phase, teens with mental health concerns had a higher weekly number of posts than teens without mental health concerns (b4 = 4.884, *p* < 0.001), but the former appeared to have a steeper declining trend then (b5 = −0.132, *p* < 0.001). However, in the post-pandemic phase, teens with mental health concerns than teens without such concerns had a more rapid increase in the weekly number of posting (b7 = 0.114, *p* = 0.001).

Similarly, Table 2 right panel shows results based on the ITSA model that predicted weekly emotion levels in the posts/comments while comparing teens with and without mental health concerns. The model has a good fit and explained 63% of the variance in the outcome variable (R-square = 0.629). Prior to the pandemic outbreak, there was a declining trend in the negative emotion (b1 = −0.002, *p* < 0.001) for teens without mental health concerns. For teens without mental health concerns, the level (interception) of negative emotions was higher during the post-outbreak phase than during the pre-pandemic phase (b2 = 0.015, *p* = 0.006), and the trend reversed from declining to increasing (b3 = 0.02, *p* < 0.001). Teens with mental health concerns had a higher level of negative emotion than teens without during the pre-outbreak phase (b4 = 0.034, *p* < 0.001), but they were not different in the trend then (b5 = −0.001, *p* = 0.598). Teens with and without mental health concerns were not significantly different in the emotion level and trend during the post-outbreak phase.

## 4. Discussion

The current study used Reddit data to examine how the COVID-19 pandemic evolution may impact teens’ psychological wellbeing as reflected in their social media engagement. We explored and described the dynamics of teens’ posting/commenting behavior and emotion expression over time. The findings showed that teens’ subreddit participation, posting/commenting frequency, and emotion expression were correlated with significant pandemic events. The study also found discrepancies in the examined indicators among teens with and without mental health concerns. We discussed the major findings in detail below.

### 4.1. Subreddit Participation Patterns

The weekly total number of active participants in the r/Teenagers subreddit was related to significant pandemic events. The major pandemic headline news, be it positive or negative, was generally followed by a hike in the number of teens posting or commenting on the r/Teenagers subreddit. For example, pandemic-related significant events, such as the WHO’s declaration of COVID-19 as a pandemic, the US declaration of it as a national emergency, and promising reports about vaccines and treatments, were accompanied by the increased number of teens participants in the related weeks on the r/Teenagers subreddit. Such a linkage is aligned with previous research that shows increasing dynamics and interplay between traditional and social media [42,43].

The findings show that the weekly number of teen Redditors on the three mental health subreddits had been generally stable throughout the year, regardless of the pandemic events. It is possible that the mental health subreddits were primarily used by people who experienced severe mental health problems, and the impact of the pandemic on mental health may be comparatively moderate and does not reach the level for their engagement in mental health subreddits. The number of active participants on the r/SuicideWatch and r/Depression subreddits declined noticeably toward the end of the year (December), which contrasted with the increase in r/Teenagers. The upcoming holiday season during the period could have increased connections with family members and friends for teens with mental health concerns, which may reduce their symptoms and subsequent engagement in the mental health subreddits.

### 4.2. Posting/Commenting Frequency Patterns

Overall, the mean weekly number of posts/comments per participant, or weekly posting/commenting frequency, for teens in the r/Teenagers subreddit was greater than that of the three mental health subreddits. The r/Teenagers subreddit is an online community for broad topics relative to the specialized communities of the three mental health subreddits, so it was rather natural that the former had a larger mean number of posting/commenting. Again, the change in the weekly posting/commenting frequency in r/Teenagers appeared to be related to significant pandemic events. In particular, after COVID-19 was declared as a pandemic by the WHO and a national emergency in the US, the mean number of weekly posting/commenting in r/Teenagers increased in the subsequent months. The mean number gradually declined from late July to mid-October. It is possible that during the summer and its extension due to delayed school opening, teens may be more likely to engage in other activities such as games or outdoor activities rather than spending time on social media interactions. Furthermore, the substantial fluctuation in r/SuicideWatch relative to other subreddits over time may suggest that teens with suicidality concerns are more subject to the pandemic’s influence (see Figure 2).

The comparison between teens with and without mental health concerns on r/Teenagers reveals interesting results. Compared with teens without mental health concerns, teens with mental health concerns (e.g., teens who ever visited any of the three mental health subreddits in the years) had a much higher mean weekly posting/commenting frequency as well as a larger fluctuation over time. Furthermore, the results based on the ITSA further indicated that although teens with and without mental health concerns both reversed the trend from declining to increasing in posting frequency after the pandemic outbreak, the former showed a larger increase. This suggests teens with mental health concerns are more susceptible to the pandemic’s influence and deserve special service attention.

Research has suggested that teens with internalizing problems are often characterized by withdrawal and other avoidance behaviors and are less likely to be active in social interactions [44]. The current findings contrast this perception, showing that teens with internalizing problems may desire social interaction no less or even more than that of peers, but fear and concern may inhibit their expression in the real world [44]. Social media provide an important platform for the social interaction needs of teens with mental health concerns. It is also important for mental health service programs to develop a friendly environment and offer responsive training to meet social interaction needs of the teens. In addition, the findings also suggest that teens with mental health concerns may be more sensitive to the pandemic events than teens without such concerns because the former showed a larger fluctuation in weekly posting/commenting frequency.

### 4.3. Emotion Expression Patterns

The findings showed that teens on the mental health subreddits expressed a more negative emotion than teens on r/Teenagers. Such findings are consistent with extant literature: teens with mental health problems are often troubled by negative emotions and more sensitive to the external environment [45,46]. Among the three mental health subreddits, the level of emotion was generally stable for r/Depression and r/SuicideWatch throughout the year, but it had a spike in week 17 for r/Anxiety and from then remained at a relatively high level for much of the year. The spike corresponded to the intense media coverage of two shooting sprees in Canada and Lebanon, which may heighten anxiety.

When comparing teens with and without mental health concerns on their posts/comments on r/Teenagers, the former had a slightly higher level of negative emotion than the latter. The ITSA further confirmed that although teens with mental health concerns showed a slight increase in negative emotions after the pandemic, this negative emotion level was not quite different from teens without mental health concerns. This suggests that teens with and without mental health concerns are somewhat similar in expressing emotions in the general online community. When comparing emotions of posts/comments across the subreddits, the negative emotion on r/Teenagers was substantially lower than that on the other three mental health subreddits. This indicates that teens with mental health concerns may be more likely to express their negative emotions in a mental health community in which people share common concerns but not in a general community. This phenomenon further suggests the importance of routinely screening teens’ mental health problems and providing them with timely support and services [47]. Otherwise, they may hide the problems from others, including friends or family members [48]. Given that many teens with mental health concerns may use social media, such as Reddit, to exchange information and support on mental health issues, it is a promising strategy to introduce trained mentors into online mental health communities to offer enhanced support and service referral for needing participants [49].

### 4.4. Limitations

Several limitations in the study should be noted. First, the current study used Reddit data to examine how the pandemic may be related to teens’ social media engagement as reflected in posting behavior and emotion expression. Future research can further focus on these posts and comments’ content more closely to advance the understanding of such linkage. Second, although Reddit is one of the most popular social media platforms used, many teens may not use Reddit; thus, the findings’ generalizability may be limited. Third, Redditors are anonymous, and there is no guarantee that those in r/Teenagers are teenagers and those in the three mental health subreddits have mental health concerns. However, as in any research using data mining techniques with social media data, an approximation is often necessary to define a sample or construct. We followed previous research to define the sample approximately [32,33,36,37]. Future research can consider using survey research with Redditors to assess to what extent participants of specialized subreddits are related to subreddits’ focuses for a better research base. Finally, although the study traced teens’ posting and emotion on Reddit for one year along with the pandemic’s evolution, it still only covered a segment of the evolving pandemic environment and may miss important more recent dynamics. For example, the recent spread of the highly infectious delta variant of the COVID-19 virus has imposed more risks to youth’s health and mental health [50]. Therefore, it is important for future research to extend the investigation to understand how this changing pandemic environment may differently affect youth social media engagement and mental health.

## 5. Conclusions

The current study is the first one that focused on teens to examine how the pandemic may affect their social media engagement and emotion expression over an extensive period. The study is also rare because it compares social media engagement in the same online community between teens with and without mental health concerns. The findings indicate that teens’ posting activities and emotions fluctuated along with the pandemic evolution, and there was a discrepancy between teens with and without mental health concerns. Across the examined subreddits, teens on r/Teenagers had an overall greater posting frequency than teens on the three mental health subreddits, but they had a substantially lower level of negative emotions. However, when social media engagement and emotion on r/Teenagers were compared between teens with and without mental health concerns, teens with mental health concerns posted/commented nearly twice as frequently than teens without mental health concerns, but the former’s negative emotion was only slightly higher than the latter. The results from the ITSA further revealed the level and trend transition of posting and emotion before and after the pandemic outbreak. The findings extend the understanding of the relationship between the pandemic environment and social media engagement in reflecting teens’ psychological wellbeing. In particular, they offer novel perspectives on the discrepancy between teens with and without mental health concerns. The findings call for more attention to the relationship between the pandemic environment and teens’ psychological wellbeing as reflected in their social media engagement, as well as the need to screen teens’ mental health problems and facilitate their social interactions by promoting a safe environment.

## Figures and Tables

**Table 1:** Sample emotional coding of posts/comments.

r/Teenagers post (0.1 0.6 0.1 0.1 0.2 0.65) Title: Kinda scared Body: I don’t normally post but I needed some way to vent. This isn’t me karma whoring because what does karma even do, no one may see this but I’m fine with that.//Recently I was diagnosed with growth hormone deficiency, I’m not sure what it exactly means, but my parents say it’s good I’ve been diagnosed because they finally know why I’ve been short my entire life and that they can treat it. I’ve heard it’s rare but possible side effects could include something like blurry vision or possible chance of seizure later in life. I’m scared and I just hope I can get through this. Thx for reading.
r/Depression comment (0.1 0.5 0.1 0.3 0.1 0.75) When you tell your father that you are suicidal have depression and severe anxiety and can’t go outside without overdosing on Atarax and he just complains “so what am I supposed to do call you ambulance?!” And I’ll probably be forced to get a job and have another mental breakdown because I can for a short time act normally therefore completing they tests so they think I can work just fine and have only 20% reduction in productivity even tho that I can’t leave home for longer than 5 min with medication.//Tried to get pension but got declined because they see nothing serious enough on me and have only 20% reduction in productivity and am only disadvantaged worker. Also don’t want to be locked up somewhere because I’m scared of it and would probably be forced to socialise with others and share room, showers, toilets all kinds of stuff, would rather kill myself than that.
r/Anxiety post (0.1 0.4 0.2 0.4 0 0.62) Title: Being extroverted and having anxiety suck Body:At school I’m extremely social and have no problem speaking to new people and making friends, but when my close friends ask me to hang out, i get extremely anxious and feel nauseous while planning, while getting ready to go out, and for a while into the hangout//These moments of anxiety overwhelm me and make me feel like staying at home is much easier, which makes no sense because i hate being alone//I stopped asking people to hang out unless they ask me because of this and it honestly just sucks.
r/SuicidWatch post (0 0.3 0 0.6 0 0.87) Body: Why can’t I be just stable and consistent, just for once? Just see myself as myself, not have constant mood swings, not being able to tell what is your indentity, personality, whatever? But like a chorus of my suicidal thoughts, there always is “but it will never happen”. And they’re right. All my nightmares have made me fear death. They affect me as if everything there happened irl. i love dreams, because they at least are a sweet desert from ghr bitter reality, but bad dreams affect me terriblt. now i look at what i just wrote and this all seems so badly written. oh well.

Note. The numbers in the first row of each cell represent the emotion ratings of anger, fear, happiness, sadness, surprise, and the compound scale, which equals anger + fear − happiness + sadness. Surprise (the number 5 value) was dropped in the compound scale. //in the texts indicates starting a new paragraph in the original posts/comments.

**Table 2:** Multi-group Interrupted Time Series Analyses of posting and emotions on r/Teenagers by comparing teens with and without mental health concerns.

Parameter	Weekly Mean Number of Posts/Comments				Weekly Emotion in Posts/Comments			
	b	Approx. SE	*t* Value	Approx.	b	Approx. SE	*t* Value	Approx.
				Pr > |t|				Pr > |t|
b0	4.948	0.039	125.520	<0.0001	0.231	0.001	217.960	<0.0001
b1	−0.028	0.009	−3.260	0.002	−0.002	0.000	−9.410	<0.0001
b2	0.741	0.226	3.280	0.002	0.015	0.005	2.800	0.006
b3	0.032	0.015	2.210	0.030	0.002	0.000	5.990	<0.0001
b4	4.884	0.127	38.330	<0.0001	0.034	0.008	4.230	<0.0001
b5	−0.132	0.024	−5.510	<0.0001	−0.001	0.002	−0.530	0.598
b6	1.512	1.007	1.500	0.137	0.011	0.010	1.070	0.289
b7	0.114	0.032	3.600	0.001	0.000	0.002	0.280	0.780
R-square	0.855				0.629			

Note. The interruption point of the time series was set in week 11 when the WHO declared COVID-19 as a pandemic. b0 = interception for non-mental health concerning teens (“NMH teens” hereafter) during the pre-pandemic outbreak (pre-outbreak hereafter) phase; b1 = slope for NMH teens during the pre-outbreak phase; b2 = interception change for NMH teens from the pre-outbreak to post-outbreak phase; b3 = slope change for NMH teens from the pre-outbreak to post-outbreak phase; b4 = difference in the interception between MH and NMH teens during the pre-outbreak phase; b5 = difference in the slope between MH and NMH teens during the pre-outbreak phase; b6 = difference in the interception between MH and NMH teens during the post-outbreak phase; b7 = difference in the slope between MH and NMH teens during the post-outbreak phase. See Caswell (2021) [41].

## Data Availability

Data are publicly available at reddit.com.
